# On the Determination of Cr(VI) in Cr(III)-Rich Particulates: From the Failure of Official Methods to the Development of an Alternative Protocol

**DOI:** 10.3390/ijerph191912111

**Published:** 2022-09-24

**Authors:** Andrea Spinazzè, Davide Spanu, Pietro Della Bella, Cristina Corti, Francesca Borghi, Giacomo Fanti, Andrea Cattaneo, William Robert Wise, Stefan John Davis, Domenico Maria Cavallo, Sandro Recchia

**Affiliations:** 1 Dipartimento di Scienza e Alta Tecnologia, Università degli Studi dell’Insubria, Via Valleggio, 11, 22100 Como, Italy; 2 Dipartimento di Scienze Teoriche ed Applicate, Università degli Studi dell’Insubria, Via Dunant, 3, 21100 Varese, Italy; 3 Institute for Creative Leather Technologies, University of Northampton, University Drive, Northampton NN1 5PH, UK

**Keywords:** chromium (VI) exposure evaluation, particulate matter, chromium speciation, ion chromatography, ICP-MS, isotope spiking

## Abstract

The goals of this work are the evaluation of the performances of official methods in the challenging determination of Cr(VI) in Cr(III)-rich particulate matter, and the development of a novel and robust analytical protocol for this issue. A liquid chromatography inductively coupled plasma mass spectrometry apparatus (LC-ICP-MS), together with an isotope-enriched spike addition technique, was used to allow the study of Cr(III)/Cr(VI) interconversions during the extraction step. An original separation strategy based on Cr(OH)_3_ head-column stacking was developed to tolerate high concentrations of Cr(III) (up to 10 mg/kg, with a Cr(VI) limit of detection of 0.51 µg/kg) without the need of any sample pretreatment. After observing, the official extraction protocols always yield false positive values in the challenging situation of particulate matter of leather industries (where huge amounts of Cr(III) are present), a new extraction strategy was developed. The novel procedure involves a 48-h extraction at room temperature using a pH-8 phosphate buffer, which demonstrated that no Cr(III)/Cr(VI) interconversions occur during this phase. To get rid of any possible interference caused by co-extracted substances, the measurement of the redox potential, together with the addition of a Fe(II)/Fe(III) redox buffer was performed to fix chromium speciation during the overall analytical protocol.

## 1. Introduction

Chromium (Cr) speciation is an extensively studied topic owing to the striking difference in toxicity of its two most stable oxidation states, the trivalent (Cr(III)) and hexavalent (Cr(VI)) forms [1,2,3]. The former is considered an essential nutrient for living organisms playing a crucial role in the metabolism of glucose and lipids and is characterized by a low toxicity profile [2]. Oppositely, Cr(VI) is classified as Group 1 (carcinogenic to humans) by the International Agency for Research on Cancer (IARC) [4], and chronic exposure to such species can cause severe damage to the entire respiratory system owing to its oxidizing power [5]. The potential risks associated with chromium exposure are critically relevant considering that workers in various industrial sectors, including electroplating, welding, painting, and the leather industry, are exposed to high concentrations of airborne chromium, mainly through inhalation and dermal contact. Dermatological conditions, renal disorders, asthma as well as lung cancer have been associated with some of these occupational exposures [6,7,8,9,10]. For a reliable risk assessment, it is, therefore, necessary to accurately quantify Cr(VI), avoiding any Cr(III)/Cr(VI) interconversion, as only hexavalent chromium (Cr(VI)) occupational exposure has to be considered within the occupational safety and health (OSH) legislation on exposure to carcinogens or mutagens at work [11,12]. In addition, the toxicology of Cr(VI) differs among a wide variety of Cr(VI) compounds, mainly depending on their solubility in water: highly insoluble chromates (e.g., ZnCrO_4_, BaCrO_4_, and PbCrO_4_) are generally considered more toxic than soluble ones as the latter are more rapidly absorbed and reduced to Cr(III) into the bloodstream of living organisms [1,13]: for this reason, it is fundamental to quantify the total Cr(VI) concentration considering both the soluble and insoluble fraction.

In the framework of OSH, the classical approach for occupational exposure assessment through environmental monitoring is based on the active personal sampling of airborne particles and subsequent analysis to determine Cr(VI) levels; exposure values are then compared to properly selected Occupational Exposure Level Values (OELVs) [14]. The recent binding Occupational Exposure Limit set under Directive (EU) 2017/2398 [15] is 10 μg/m^3^ (8-h time-weighted average (8-h TWA)) until January 17, 2025; after that period, the OELV (8-h TWA) will be limited at 5 μg/m^3^. Some countries have proposed and adopted other (sometimes more stringent) OELVs [6,16].

Up to now, the official methods proposed for the measurement of total Cr(VI) in airborne particulate matter (PM) are the ISO 16740:2005 standard, NIOSH 7600-7605, and OSHA ID-215 [17,18]. The three protocols are quite similar as they all exploit (i) polyvinylchloride (PVC) filters as PM collectors, (ii) a 1,5-diphenylcarbazide (DPC) based colorimetric method for Cr(VI) detection (direct or after a chromatographic separation), and (iii) alkaline extractants operating at 95 °C (2% NaOH-3% Na_2_CO_3_ for ISO 16740 and NIOSH 7605, while 10% Na_2_CO_3_-2% NaHCO_3_ mixed with phosphate buffer and magnesium sulfate for OSHA ID-215) [17]. Although these methods are designed to obtain the selective leaching of total Cr(VI), it was shown that they are not sufficiently robust, as problems related to the occurrence of Cr(III) to Cr(VI) interconversion during the hot alkaline extraction are reported [19]. Both these two issues (the utilization of basic conditions and of hot extraction) were shown to be problematic even when considered separately: the utilization of hot extraction induces Cr(III) oxidation even working in acid conditions (that are known to favor the opposite reaction) [20], in addition, the utilization of basic conditions induces Cr(III) oxidation even if the extraction is performed at room temperature [21]. Any Cr(III) oxidative process during extraction should be avoided as it can clearly provide Cr(VI) overestimations, and this problem is especially critical for all particulate matters where the amount of Cr(III) may be markedly higher than Cr(VI) [22,23]. In this context, the leather factory environment could represent one of the most challenging situations for any given analytical protocol as Cr(III) is used in huge amounts [24,25,26]. Cr-tanned leathers contain up to 3–5% Cr(III) [24,25], which means very high chromium concentrations are expected if the indoor particulate arises mainly from leather shavings. The absence of Cr(VI) in leathers containing particulate cannot be claimed by simply stating that only Cr(III) is used during the tanning treatment, as it is well known that during the aging of leathers, partial oxidation of Cr(III) may occur [26].

From all the above issues and taking into account the weaknesses of the official methods and the absence of any standard reference material, it is clear the availability of a proper tool to carefully monitor for any Cr(III)-Cr(VI) interconversion during extraction is indeed necessary. This tool would be essential to validate data or to revise any analytical protocol being proposed. The speciated isotope dilution mass spectrometry (SIDMS), represents such a tool that can be used to monitor and (sometimes) correct for Cr(VI)–Cr(III) interconversions [27,28,29,30,31]: within the SIDMS technique ^53^Cr and ^50^Cr enriched isotopes can be spiked to correct the quantification using isotopic abundance ratios [27,28,29,32].

Under this framework, we decided first to develop an analytical method based on liquid chromatography inductively coupled plasma mass spectrometry (LC-ICP-MS), to gain improved sensitivities with respect to traditional colorimetric methods [20,21,33] and to allow for the monitoring of the Cr(III)/Cr(VI) interconversion processes by speciated isotope dilution [31,34]. In parallel, it was necessary to develop a novel separation strategy, since the commonly employed Cr(III)/Cr(VI) LC separation methods cannot bear the presence of huge amounts of Cr(III) [33,35]. This developed method was subsequently used to confirm the suspected failure of the official methods in trying to yield reliable Cr(VI) concentrations in the particulate matter sampled in different leather industries.

To fix the problems encountered with the official methods A novel extraction protocol for the total Cr(VI) determination was then developed starting from the ISO 17075 method for the determination of Cr(VI) in leather [36]. According to the literature relating to the stability of Cr species at varying pH [19,37,38], a long extraction at room temperature at circumneutral pH was optimized. 

Finally, the goodness of the overall protocol was evaluated in the case study of particulate matter sampled in leather industries and used to properly characterize the risk of occupational exposure to Cr(VI).

## 2. Materials and Methods

### 2.1. Reagents

Ultrapure water was used for the preparation of each solution used and was produced with a Sartorius Arium mini plus UV Lab Water System. Ultra-pure H_3_PO_4_ (Merck, Milan, Italy, Suprapur 85% in water) and K_2_HPO_4_ (Carlo Erba, Milan, Italy, ≥98% pure) were used for the preparation of the ISO extracting solution: 1:10 m/m diluted ultra-pure H_3_PO_4_ was also used to neutralize NIOSH extracting solutions. NaOH (Sigma Aldrich, Milan, Italy, 99.99% pure) and Na_2_CO_3_ anhydrous (Sigma Aldrich, Milan, Italy, ≥99.5%) were used for the preparation of the NIOSH extracting solution. NH_4_NO_3_ (Sigma Aldrich, Milan, Italy, ≥99.0%) and NH_3_ (Sigma Aldrich, Milan, Italy, 25% in water) were used for the preparation of the mobile phase.

The redox buffer was prepared by dissolving 4.30 g of Potassium Hexacyanoferrate(II) 3-hydrate (K_4_[Fe(CN)_6_]·3H_2_O, Merck, Milan, Italy, ≥99% pure) and 3.30 g of Potassium Hexacyanoferrate(III) (K_3_[Fe(CN)_6_], Carlo Erba, Milan, Italy, ≥99% pure) in 1 L of ultrapure water.

The concentrated 1000 mg/L Cr(VI) standard solution was obtained by dissolving 2.829 g of K_2_Cr_2_O_7_ (Carlo Erba, Milan, Italy, ≥99% pure) in 1L of ultrapure water. Starting from this concentrated solution, all other Cr(VI) standards were obtained by proper dilution.

The concentrated 1000 mg/L Cr(III) standard solution was obtained by dissolving 7.692 g of Cr(NO_3_)_3_]·9H_2_O, (Merck, Milan, Italy, ≥99% pure) in 1L of ultrapure water. Starting from this concentrated solution, all other Cr(VI) standards were obtained by proper dilution.

Solid PbCrO_4_ was obtained by precipitation with an excess of Pb, mixing equal parts of a 1.5 M solution of Pb(NO_3_)_2_ (Sigma Aldrich, Milan, Italy, ≥99%) with a 0.5 M solution of K_2_Cr_2_O_7_. The precipitate was washed several times with ultrapure water to remove any soluble residual.

A 10 mg/L vanadium standard solution was obtained by dilution of a 1000 mg/L V standard solution (Fluka, Milan, Italy, TraceCERT^®^).

Both the ^50^Cr(VI)-enriched standard solution (10 ± 0.4 µg/g) and the ^53^Cr(III)-enriched standard solution (10 ± 0.2 µg/g) were purchased by ISC science (Oviedo, Spain). The certified isotopic abundances, along with the ones of naturally occurring chromium are reported in Table 1.

**Table 1:** Certified isotopic abundances (%) for the ^50^Cr(VI)-enriched and the ^53^Cr(III)-enriched standard solutions. For clarity, isotopic abundances of naturally occurring Cr are also reported.

Standard Solution	^50^Cr (%)	^52^Cr (%)	^53^Cr (%)	^54^Cr (%)
^50^Cr(VI)-enriched std	93.7	5.7	0.51	0.10
^53^Cr(III)-enriched std	<0.1	2.55	97.3	0.11
naturally occurring Cr	4.345	83.789	9.501	2.365

### 2.2. Particulate Sampling

Workers may be exposed to Cr(VI) via inhalation and dermal exposure; however, for the purpose of the study, the latter was not considered. Personal air samples of the inhalable fraction of airborne particles were collected in the breathing zone of selected workers for the assessment of inhalation exposure [39]. Sampling was performed at a flow rate of 2 L/min with a two-piece poly-propylene cassette filter-holders (SKC Inc. 863 Valley View Road Eighty Four (PA), USA) fitted with pre-weighed PVC filters (PALL-GLA-5000 low-ash PVC membranes; 37 mm in diameter; porosity 5 µm). Furthermore, inhalable particles were also monitored by fixed-site sampling in different spots of the working area; these sampling lines were placed at the same time and place of personal sampling. Workers involved in the shaving of tanned leathers were chosen because of their expected high exposure to particulate matter. Each sampling campaign consisted of both personal sampling and fixed-site sampling: 12 pairs of samples collected in parallel (duplicates) through personal monitoring were obtained in four different shaving areas of two leather factories. Monitored workers were divided into two “Similar Exposure Groups” (SEGs), according to EN 689 [14]. The first (SEG A) and second (SEG B) SEGs included workers involved in leather shaving and enrolled in the first and second companies under investigation, respectively. Further, four pairs of duplicates from fixed-site environmental sampling were collected contextually. These samples were taken to cover all the most relevant situations, that is, the particulate matter emitted from shaving of bovine, swine, goat, and chromium-free tanned leathers. The air samples were first analyzed gravimetrically to determine the inhalable particle fraction: in all cases, the determined values, which are reported in the Supplementary Information, are far below the exposure threshold values (10 mg/m^3^). After that, the samples were analyzed for total Cr(VI) using both the NIOSH 7600 protocol and the modified ISO 17075 protocol. Results of exposure monitoring were used for risk characterization, performed by testing compliance with the European OELV for Cr(VI). The compliance with OELV was statistically evaluated by comparing it with the upper confidence limit (UCL) of 70% with the 95th percentile of the distribution of at least six measurements. If the UCL is lower than the OELV, it is concluded that the probability of exceeding the OELV is acceptably low, that is a condition of compliance with the limit value. Further details on exposure assessment and risk characterization methods are reported in Supplementary Materials.

### 2.3. Extraction According to the NIOSH 7600 Protocol

The extraction solution for the NIOSH method was prepared by dissolving 20 g of NaOH and 30 g of Na_2_CO_3_ anhydrous in 1 L of ultra-pure water and controlling that the pH of this solution is above 12. This solution was then degassed for 5 min with N_2_ before every extraction. All extractions were performed at 95 °C (ensured with water bath) for 45 min, using 5 mL of extractant in a 25 mL Schlenk tube, where the PVC filter was previously inserted. Before starting the extraction, 50 µL of the ^50^Cr(VI)-enriched standard solution and 50 µL of the ^53^Cr(III)-enriched standard solution were added to the Schlenk tube. During the extraction, the Schlenk tube was constantly purged with Ar and gently swirled every 10 min. After extraction, the solution was cooled down and transferred into a 30 mL Low-Density Polyethylene (LDPE) container (Nalgene^®^): a 1:10 solution of ultra-pure H_3_PO_4_ was added to lower the pH to 8.0 ± 0.1. 250 µL of the 10 mg/L V standard solution is then added to the transferred and neutralized sample, to obtain a V concentration equal to 100 µg/kg after dilution to 25.0 g.

### 2.4. Extraction According to the Modified ISO 17075 Protocol

The extraction solution for the modified ISO method was prepared by dissolving 22.8 g of K_2_HPO_4_·3H_2_O in a 1L volumetric flask with approximately 800 mL of ultra-pure water and adjusting the pH to 8.0 ± 0.1 with ultra-pure concentrated H_3_PO_4_: after pH adjustment, the solution was made up to volume with ultrapure water. In contrast with the ISO protocol, we found it unnecessary to perform any degassing operation. To perform the extraction the filter was placed in a 30 mL LDPE container with approximately 5 g of extraction solution. Then, 20 µL of the ^50^Cr(VI)-enriched standard solution and 20 µL of the ^53^Cr(III)-enriched standard solution, together with 100 µL of the 10 mg/L V standard solution were added to the LDPE container and the weight was raised up to 10.0 g with the extraction solution. The extraction was then performed with an orbital shaker (180 rpm) for 48 h. Whenever specified in the text, 200 µL of the Fe(II)/Fe(III) redox buffer was added to the LDPE before raising the weight to 10.0 g.

### 2.5. Oxidation-Reduction Potential (ORP) Measurements

An AMEL model 337 potentiometer equipped with a combined Platinum electrode was used for all ORP measurements reported in this work. These electrochemical measurements were performed at different stages of and at fixed times after the extraction procedures to monitor the evolution of the redox conditions in the extracting solutions and to assess the effect of the Fe(II)/Fe(III) redox buffer, when present.

### 2.6. Chromatographic Separation and ICP-MS Detection

The chromatographic system is composed of a Metrohm 709 IC PEEK Pump equipped with a Rheodyne 9725 injection valve (a 64 µL homemade PEEK sample loop was always used) and by a Hamilton PRP-X100 (25 cm x 4 mm i.d.) anionic column, which is directly connected to the nebulizer of the ICP-MS (Thermo, ICAP Q). The optimized mobile phase is composed of a 20 mM NH_4_NO_3_ solution at pH 9.5, which is prepared as follows: 1.6 g of NH_4_NO_3_ are dissolved in 1 L of ultrapure water and sonicated for 20 min; the pH is then adjusted to 9.5 ± 0.05 with the 25% NH_3_ solution. All samples were filtered with a disposable 0.25 µm membrane filter just before injection, while Cr(VI) elution is performed in isocratic conditions at 0.8 mL/min.

The ICP-MS is operated in the Kinetic Energy Discrimination (KED) mode, using He as collision gas and with all tuning parameters set following the manufacturer’s indications. The *m/z* channels 50, 51, 52, 53, 54, and 56 were followed, setting a dwell time equal to 20 ms for all channels. 

### 2.7. Natural Cr(VI) and Isotopic 50Cr(VI)-53Cr(III) Spikes Quantifications

From Table 1, both natural Cr and isotopically enriched standards have significant amounts of chromium for all the 50, 52, 53, and 54 masses. Therefore, a simple direct univariate “one mass-one species” correlation cannot be established in this case. Rather, every species contributes to each *m/z* signal in a different but additive way. To determine the concentrations of naturally occurring Cr(VI) present in samples (from now to on named as natural Cr(VI)), together with the concentrations of the added ^50^Cr(VI) and ^53^Cr(III) added spikes, the procedure reported in the Supplementary Materials was used.

### 2.8. Quality Assurance and Quality Control of the Developed Protocol

Since no Cr(VI) PM Standard Reference Materials (SRMs) are commercially available, a direct quality assurance/quality control protocol performed on certified specimens is not feasible. Nevertheless, the utilization of the SIDMS technique, which is a primary method of measurement in chemical analysis [40], together with the reasons explained in Section 3.5, are more than sufficient to state that the developed method is surely accurate. For the NIOSH 7600 method, the precision was evaluated by performing three replicated measurements for each of the four samples reported in Table 2: the calculated pooled relative standard deviations for natural Cr(VI) and ^50^Cr(VI) (2.32% and 2.14%, respectively) are not significantly different. Slightly better precisions (1.78%) were attained for the modified protocol using the same strategy above reported, but on the data of Table 3: this evidence is probably related to the simplified sample workup of the developed method.

Quality control was performed by adopting the following procedures. The LC-ICP-MS instrument was calibrated on a daily basis, by analyzing three standard solutions spiked with 20 μg/kg of ^50^Cr(VI) and ^53^Cr(III), which contained 0, 50, and 200 μg/kg of natural Cr(VI), respectively. In all extraction sections, a blank filter was also extracted and analyzed to control for eventual unwanted reagents or glassware contaminations. 

Finally, it should be noted that the SIDMS technique (which was used for each sample) is intrinsically a very robust quality control technique, as it certifies the presence of false positive/false negative data.

## 3. Results

### 3.1. Optimization and Features of the Chromatographic Separation

The presence of high quantities of Cr(III) in the extracted solutions is a relevant issue not only in terms of its possible oxidation to Cr(VI) but also for the potential overlapping of the Cr(III) peak tail (which is very pronounced at high Cr(III) concentrations) with the Cr(VI) peak. It should be noticed that this latter problem is strictly related to the utilization of ICP-MS as the detector, since the conventional UV-Vis detection after post-column derivatization with DPC cannot detect Cr(III). In principle, to solve this problem, one possible strategy relies on the inversion of the elution order, that is, having Cr(VI) exiting first from the column. However, this cannot be accomplished by using a cationic column instead of the normally used anionic one, because Cr(III) is present in various forms ranging from cationic to neutral and/or anionic [19,33]. An interesting alternative approach consisting of the complexation of Cr(III) with EDTA and the derivatization of Cr(VI) with DPC over an anionic column was recently reported [31,41]. In this case, the elution order is reversed because the former complex is anionic while the second is cationic. Considering that to reach the quantitative complexation with EDTA, it is necessary to heat at 70 °C for at least 25 min, this approach was not followed to avoid any possible Cr(III)/Cr(VI) inter-conversion that, as already mentioned, is boosted by heating.

We, therefore, decided to solve this problem by exploring the possibility of a quantitative head-column stacking of Cr(III) as Cr(OH)_3_. This is an unusual LC strategy because the accumulation of a precipitate is expected to gradually clog the column with the increasing the working pressure of the LC system. As will be clear later, this does not happen under the conditions that we have optimized.

**Figure 1:**
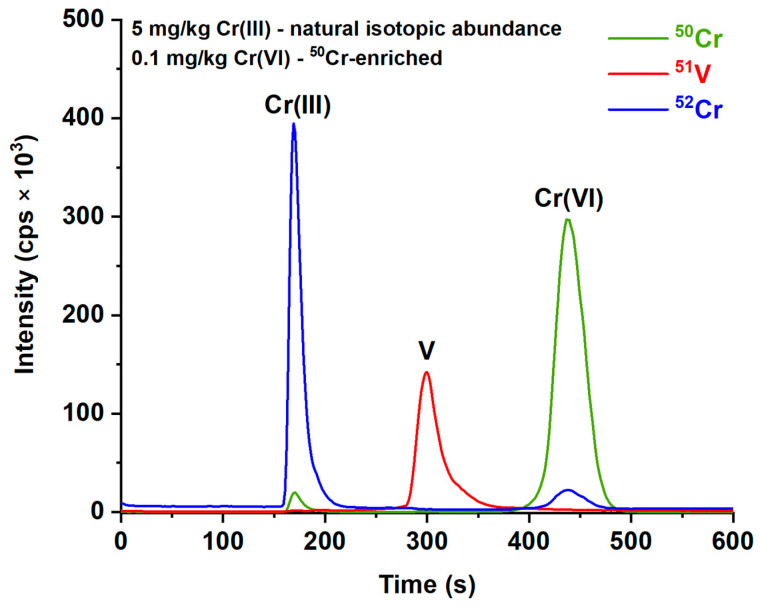
Chromatographic profiles obtained in optimized conditions for a 5 mg/kg Cr(III) + 100 ppb ^50^Cr(VI) standard solution, with 100 ppb of Vanadium used as internal standard. Green, red and blue profiles represent the signals recorded at *m/z* 50, 51, and 52, respectively.

According to Palmer et al. [42], the dominant form of Cr(III) is Cr(OH)_3_ when the pH is between 8 and 11, while the formation of soluble Cr(III) hydroxometallates is favored at more basic pH values. For this reason, we tried different mobile phases within the 8–11 pH range (varying both the pH and the NH_4_NO_3_ concentration) finding that working at pH = 9.5 with a 20 mM solution of NH_4_NO_3_, it is possible to retain the most Cr(III) with only a minor part eluting from the anionic column with all the cations. It should be recalled that NH_4_NO_3_ was chosen because it fully decomposes in the ICP torch, leaving no residues on the extraction and skimmer cones. The chromatographic separation obtained in the optimized conditions is reported in Figure 1, where it can be seen that the residual cationic Cr(III) is sufficiently small to be very well separated from the Cr (VI) peak, even working with a 5 mg/kg Cr(III) solution.

The limit of detection (LOD) under these separation conditions, determined according to the IUPAC definition and European guidance [43] by injecting 10 times a 3 µg/kg Cr(VI) solution, is equal to 0.51 µg/kg: considering a sampled air volume of 400 L and the volume of the extractant used for the optimized protocol (10 mL), this yields a 0.013 µg/m^3^ detection limit (i.e., a 0.039 µg/m^3^ limit of quantification, LOQ) that is fully satisfactory if compared to the actual (10 µg/m^3^) and to the incoming (5 µg/m^3^) OELV set under Directive (EU) 2017/2398. The linearity was checked up to 1000 µg/kg: higher values were not tested out of the absence of any practical interest.

**Figure 2:**
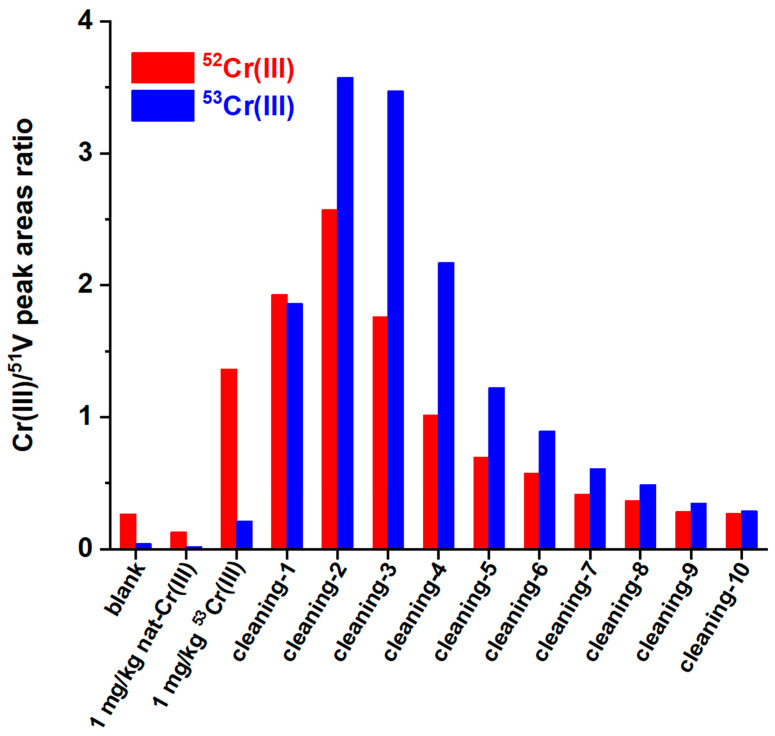
^52^Cr(III)/^51^V and ^53^Cr(III)/^51^V peak area ratios of a sequence (from left to right) of various blank and standards injections: areas determined on the cationic Cr(III) peak (first peak in Figure 1).

No column clogging was observed over hundreds of injections because we noticed that the phosphate buffer, which is present in all the injected solutions, causes a gradual dissolution of Cr(OH)_3_ with the formation of a cationic Cr(III) phosphate (probably CrHPO_4_^+^). This was verified by injecting first a 1 mg/kg standard solution of natural Cr(III), followed by a 1 mg/kg ^53^Cr(III) isotopic enriched solution, and then by several blank phosphate buffer injections. As it can be seen in Figure 2, the peak area isotopic ratio of the cationic Cr peak of the second injection (1 mg/kg of ^53^Cr(III)) is not consistent with the expected one, meaning that ^53^Cr(III) is retained by the column. Both the 53/51 and the 52/51 peak area ratios gradually increase in the subsequent blank phosphate buffer injections, meaning that both the natural Cr and the ^53^Cr(III), retained by the column, are gradually solubilized.

In conclusion, this quite uncommon and self-regenerating separation approach fully meets our requirements, i.e., the robust determination of Cr(VI) in the presence of much higher amounts of Cr(III). Moreover, the employment of ICP-MS allows the utilization of the speciated isotope addition strategy, which is fundamental to study the performance of the extraction protocols.

### 3.2. Evaluation of the NIOSH 7600 Protocol

Four different samples from fixed-sited sampling performed in four different shaving areas of two leather factories were analyzed according to the NIOSH 7600 protocol.

After sampling and before starting the extraction, each filter was spiked with a ^50^Cr(VI) and a ^53^Cr(III) standard solution to have a final concentration of the extracts equal to 20 µg/kg for each standard. All the extractions were conducted following strictly the NIOSH protocol: the only exception is represented by the utilization of Ar instead of N_2_ as protective gas, which, due to its higher density, guarantees better shielding against oxygen diffusion. As the anionic column cannot tolerate the extremely basic pH of the NIOSH extractant, it was necessary to lower the pH to 8.0 with pure H_3_PO_4_ after extraction.

In Table 2, the results obtained for the environmental sampling of the four different shaving areas are reported. Even if all data are well below the OELV, and the ^50^Cr(VI) is almost quantitatively recovered, the most problematic issue comes from the percentage of oxidation of the ^53^Cr(III) added spikes. In all extractions, we observed ^53^Cr(III) to ^53^Cr(VI) conversions ranging from 55% to 60%.

**Table 2:** Cr(VI) airborne concentrations detected with the NIOSH extraction protocol (fixed-site sampling in four different shaving areas). Data of spikes recoveries/oxidations, together with three blank extractions are presented. ORP values determined after extraction and neutralization.

Sample ID	Cr(VI) Conc. (µg/m^3^)	^50^Cr(VI) Spike Recovery (%)	^53^Cr(III) Spike Oxidation (%)	ORP (mV vs. Ag/AgCl)
Tanned goat leather shaving	0.347	91.0	60.3	180
Tanned swine leather shaving	0.626	90.1	59.4	180
Tanned bovine leather shaving	0.545	96.9	59.5	182
Tanned Cr-free leather shaving	0.282	94.6	54.8	173
Blank extraction + 20 µg/kg ^53^Cr(III)	<0.05 μg/kg *	--	68.0	190
Blank extraction + 200 µg/kg ^53^Cr(III)	<0.05 μg/kg *	--	20.2	185
Blank extraction + 1000 µg/kg ^53^Cr(III)	<0.05 μg/kg *	--	6.1	183

* In the case of the analysis of standard solutions, data refer to the concentrations found in extraction solutions.

The cause of such a relevant oxidation cannot be ascribed solely to the presence of oxidants eventually co-extracted from the particulate matter, as the same behavior is observed even in blank extractions (see the three last rows in Table 2): this would indicate a major role of residual oxygen and/or the so-called reactive oxygen species [44]. The percentage of Cr(III) oxidation is not constant with increasing Cr(III) concentrations, suggesting that this process is probably constrained by the limited availability of oxidants in the extraction solution. Independently from a deep understanding of the real nature of such an oxidation, which is out from the goals of this work, these data (and also the data that will be reported later) clearly indicate that the NIOSH protocol systematically overestimates the presence of Cr(VI) if significant amounts of Cr(III) are present in particulate matter. From the data of the last three rows of Table 2, it is reasonable to think that the extent of such an overestimation may be shadowed in all particulate samples where the real Cr(VI)/Cr(III) ratio is high and/or when both Cr(VI) and Cr(III) concentrations are high. In such cases, the official NIOSH 7600 extraction could provide reliable data, provided that any redox role played by coextracted substances can be ruled out.

Unfortunately, the addition of the ^53^Cr(III) spike cannot be helpfully used to correct such a positive bias because the amount of Cr(III) in the sample is not known and all the added ^53^Cr(III) spike is ready to be oxidized from the beginning of the extraction, while Cr(III) in the particulate is expected to be solubilized in a gradual, but unknown way.

Even if the data collected over chromium isotopic spikes are not useful to correct this bias, the utilization of isotopic spikes is fundamental to develop an extraction protocol that is able to extract Cr(VI), avoiding any Cr(III)/Cr(VI) interconversion.

### 3.3. Development of the Alternative Extraction Procedure

**Figure 3:**
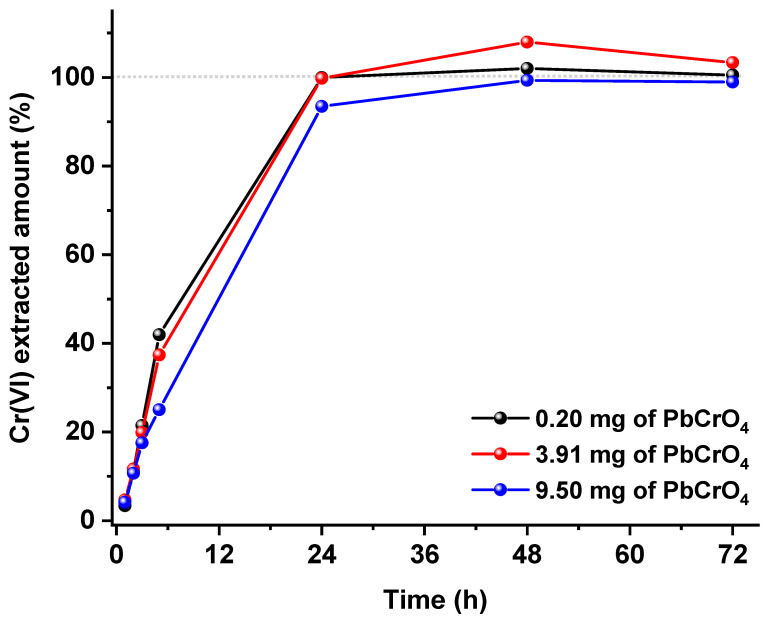
Extraction profiles of PbCrO_4_ at room temperature with 50 mL of the ISO phosphate buffer: the three curves refer to three independent extractions carried out with increasing PbCrO_4_ loadings.

As already mentioned in the introduction, the kinetics of the Cr(III)/Cr(VI) interconversion are quickened by high extraction temperatures and by the utilization of acid or basic environments, and therefore, such conditions should be avoided. Among the extractants proposed in the literature to recover Cr(VI) from various matrixes, the one used in the validated ISO 17075 method for leather samples is quite interesting and should also be a good candidate for particulate matter, as it works at room temperature with a phosphate buffer at pH 8.0. The only negative aspect of this method relates to the evidence that the insoluble Cr(VI) fraction is not extracted quantitatively. From a thermodynamic point of view, the phosphate buffer is expected to solubilize all insoluble chromates since all the respective phosphates are much more insoluble than chromates. However, the kinetics of such dissolutions could be quite slow, as they are related to the dissolution of a precipitate with the formation of a precipitate. Starting from these considerations (and since no data are reported in the literature about this kind of dissolution), we decided to study the kinetics of the extraction of the least soluble of the chromates, that is, PbCrO_4_ (K_sp_ = 1.8 × 10^−14^) with the phosphate buffer described in the ISO 17075 method. The goal was to observe if an extension of the established extraction time is sufficient to obtain total dissolution. Figure 3 depicts the kinetic extraction profiles obtained for three different PbCrO_4_ loadings. As it can be seen, in all cases, the complete dissolution is obtained after 48 h, while only approx. 20% of the insoluble Cr(VI) is extracted within the 3 h prescribed by the ISO method.

Once it is confirmed that no Cr(III)/Cr(VI) interconversions occur within such a long extraction time, this evidence opens the possibility to use such mild conditions for the recovery of the total Cr(VI) content.

The performances of the extended ISO extraction were then tested on the same environmental particulate sampling (collected in double) reported in Table 2. Even if from Figure 3 it could be argued that it is not necessary to wait for 48 h to gain the total insoluble Cr(VI) extraction, we decided to use this time for practical purposes.

The data reported in Table 3 clearly indicate the total recovery of the ^50^Cr(VI) spike, with no oxidation of the ^53^Cr(III) spike, thus confirming that Cr(VI)/Cr(III) interconversion did not happen during the extraction. Interestingly, all the data reported in Table 3 were obtained without any previous outgassing of the extraction solution, nor working under an inert atmosphere, thus simplifying the overall extraction procedure.

**Table 3:** Cr(VI) airborne concentrations detected with the ISO-modified extraction protocol (same location and time span of samples reported in Table 2). ORP values determined after extraction.

Sample ID	Cr(VI) Conc. (µg/m^3^)	^50^Cr(VI) Spike Recovery (%)	^53^Cr(III) Spike Oxidation (%)	ORP (mV vs. Ag/AgCl)
Tanned goat leather shaving	<0.013	98.1	<2.5	132
Tanned swine leather shaving	<0.013	98.8	<2.5	120
Tanned bovine leather shaving	<0.013	98.6	<2.5	131
Tanned Cr-free leather shaving	<0.013	99.9	<2.5	111

### 3.4. On the Role of Co-Extracted Substances

During the extraction, it is possible that substances other than Cr(VI) (which are present in the particulate matter) can be eventually co-extracted. Even in a restricted field such as the leather industries, the nature of the possible co-extracted substances is so wide that is completely meaningless to assess and quantify their presence and, more importantly, their possible action in terms of Cr(III)/Cr(VI) interconversion. Rather, the extraction protocol should be sufficiently robust to be not influenced by their eventual presence.

In this context, we decided to take advantage of the utilization of ORP measurements as an indicator of the presence of potential oxidation/reduction issues: high/low ORP values would indicate a potential instability of redox couples (like the Cr(III)/Cr(VI) one) towards oxidation/reduction phenomena, respectively. As a starting point, we have observed the ORP value of the phosphate buffer used for the developed extraction is equal to 256 mV (vs. Ag/AgCl) and that diluted Cr(III) and Cr(VI) standard solutions built using such a buffer are indefinitely stable since no interconversions are observed up to 2 months from their preparation. This is not surprising considering that from the Pourbaix diagram [19], such potential is nearly at the turn of the Cr(III) and Cr(VI) stability regions. Moreover, we observed that the composition of all extracts reported in Table 3 and in Table 2 (in this latter case, after neutralization) is indefinitely stable, meaning no Cr(III)/Cr(VI) interconversions occur in the ORP range between 110 and 256 mV.

On the other hand, a marked reduction of Cr(VI) was observed in all personal sampling where the measured ORP value after extraction was below 100 mV (see Table 4), indicating that, in such cases, the coextracted substances cause a negative bias in Cr(VI) quantification.

To fix this problem, we decided to use the addition of a redox buffer to the extraction solution in order to stabilize the ORP value within the stability range reported above. For this purpose, we used the Fe(CN)_6_^3−^/Fe(CN)_6_^4−^ redox buffer (see the experimental section for more details), which is able to stabilize the ORP around 220 mV. The effectiveness of this procedure was by performing an extraction directly on 250 mg of a particularly reducing leather sample, which, after extraction, showed an ORP value of 73 mV. If the same extraction is performed in the presence of the redox buffer, the ORP value remains almost constant around 220 mV and, more importantly, no Cr(VI) reduction was observed.

With this evidence, we recommend the introduction of this redox buffer into the phosphate buffer solution in all cases where the ORP values are low and/or when the isotopic Cr spikes indicate a Cr(VI) reduction. The only drawback of the utilization of the Fe(CN)_6_^3−^/Fe(CN)_6_^4−^ buffer is that it is not compatible with the conventional DPC post-column derivatization, since we have observed that the Fe(CN)_6_^4−^ species strongly interfere with the formation of the Cr(III)-DPC complex, which is the species detected by the UV-Vis detector [45]. Therefore, the Fe(CN)_6_^3−^/Fe(CN)_6_^4−^ buffer can be used only if ICP-MS is used as the detector.

### 3.5. On the Validation of Developed Extraction Protocol

No standard reference material for Cr(VI) in the particulate matter of the leather industry is commercially available, but, even if it was available, the certified value should raise doubts if the NIOSH 7600 method was used to assess the Cr(VI) concentration. Consequently, there is no way to perform a direct validation against a reference material. Nevertheless, the data so far reported are more than sufficient to assert that reliable data can be obtained with our developed protocol. First of all, The extraction protocol is equal to the validated ISO 17075 procedure for the determination of Cr(VI) in leather samples, but utilizes a longer extraction time, which is more than sufficient to extract the insoluble Cr(VI) fraction. Secondly, considering the ISO17075 method is set up to work with 6 mm × 6 mm leather pieces, no diffusional limitations are expected during the extraction of Cr(VI) from particulate particles, which are definitely much smaller. The utilization of the ^53^Cr(III) and ^50^Cr(VI) isotopic spikes revealed that no Cr(III)/Cr(VI) interconversion occurs during the long extraction. As will be shown later in real personal particulate sampling, it is fundamental to use such isotopic spikes for all sample extractions in order to verify that eventually, co-extracted substances do not cause Cr(III)/Cr(VI) interconversions. With this strategy, every single result becomes self-consistent. Finally, the ORP measurement, together with the addition of a Fe(II)/Fe(III) redox buffer, may be used to assess and correct issues related to the presence of co-extracted reductants or oxidants.

### 3.6. Cr(VI) Determination on Personal Sampling, Occupational Exposure, and Risk Characterization

An overall synopsis of the exposure monitoring results is shown in Table 2 and Table 3 (airborne Cr(VI) concentrations; fixed-site samplings) and in Table 4 and Table 5 (exposure to Cr(VI); personal samplings). Airborne Cr(VI) levels and occupational exposure levels were always lower than the OELVs (with a precautionary approach, here we consider the more stringent limit set by the European Directive—i.e., 5 µg/m^3^), regardless of the type of sampling and the method of analysis. Fixed-site samples analyzed through the NIOSH protocol (Table 2) showed that airborne Cr(VI) concentrations in the investigated companies were one order of magnitude lower than the OELV of 5 µg/m^3^. Samples collected at the same time as the latter (duplicates) but analyzed with the ISO-modified extraction protocol all resulted in <LOD values (Table 3), which were lower by another order of magnitude.

With regards to the personal exposure measurements carried out on SEG A and SEG B workers, for the samples analyzed with the NIOSH protocol (Table 4), no exposure values lower than the limit of quantification were identified.

Once the validity of each measurement was ensured (Appendix E of the EN 689 standard), it was verified that the definition of the SEG complied with adequate criteria of quality, representativeness, and number. It was verified the exposure data of each SEG are distributed with log-normal distribution (Kolmogorov–Smirnov; *p* < 0.05). It was therefore possible to apply the statistical test for comparison with the selected OELV, as described above (Appendix F of the EN 689 standard). Further, for the purpose of defining the maximum time interval before a subsequent evaluation, the indication provided by Appendix I of the EN 689 standard was applied. Table 6 shows the result of the VLEP compliance test for personal exposure to Cr(VI) by considering samples treated through the NIOSH protocol. As anticipated, no exceedances of the limit value are observed and the compliance with the selected OELV for Cr (VI) was statistically evaluated by comparing the OELV with the UCL of 70% with the 95th percentile of the distribution of measurements obtained within each SEG. Since the UCL is lower than the OELV for both the workers’ groups, the probability of exceeding the OELV is acceptably low, and therefore the risk associated with this occupational exposure can be considered acceptable for both the considered SEGs.

**Table 4:** Cr(VI) occupational exposure detected with the NIOSH extraction protocol through personal sampling in two SEGs of tanned leather shavers. Data of spikes recoveries/oxidations are presented, together with ORP values determined after extraction and neutralization.

SEG	Sample ID	Cr(VI) Conc. (µg/m^3^)	^50^Cr(VI) Spike Recovery (%)	^53^Cr(III) Spike Oxidation (%)	ORP (mV vs. Ag/AgCl)
A	A1	0.950	94.9	53.2	168
	A2	0.305	94.0	56.6	161
	A3	0.404	97.4	56.0	188
	A4	0.548	95.7	49.6	149
	A5	0.645	94.2	62.5	178
	A6	0.428	94.9	56.7	170
B	B1	0.304	98.0	64.3	176
	B2	0.514	96.0	61.8	160
	B3	0.535	97.8	63.7	173
	B4	0.394	95.1	59.3	138
	B5	0.878	94.3	57.9	144
	B6	0.326	93.0	59.8	152

**Table 5:** Cr(VI) occupational exposure detected with the ISO-modified extraction protocol through personal sampling in two SEGs of tanned leather shavers (same workers, location, and time span of samplings of Table 3). Data of spikes recoveries/oxidations are presented, together with ORP values determined after extraction.

SEG	Sample ID	Cr(VI) Conc. (µg/m^3^)	^50^Cr(VI) Spike Recovery (%)	^53^Cr(III) Spike Oxidation (%)	ORP (mV vs. Ag/AgCl)
A	A1	<0.013	98	<2.5	106
	A2	<0.013	99.1	<2.5	173
	A3	0.015	72.5	<2.5	84
	A4	<0.013	79.2	<2.5	96
	A5	<0.013	99.1	<2.5	197
	A6	<0.013	99	<2.5	130
B	B1	<0.013	99.6	<2.5	115
	B2	<0.013	98.8	<2.5	104
	B3	0.024	100.3	<2.5	122
	B4	<0.013	82.7	<2.5	97
	B5	0.014	85.9	<2.5	99
	B6	<0.013	99.1	<2.5	199

**Table 6:** OELV compliance test for Cr (VI) occupational exposure—Samples treated with the NIOSH extraction protocol (data in Table 4). GM: geometric mean; GSD: geometric standard deviation.

SEG	SEG-A	SEG-B
Number of valid measurements (n)	6	6
GM (µg/m^3^)	0.511	0.460
GSD (µg/m^3^)	1.49	1.48
OELV (µg/m^3^)	5	5
Ur	5.720	6.099
Ut (for n = 6 measurements)	2.187	2.187
Judgment	Compliance	Compliance
Period before next monitoring	36 months (J = 0.244)	36 months (J = 0.236)

Most of the samples from personal sampling analyzed with the ISO-modified extraction protocol (Table 5) are lower than the LOD (5 out of 6 for SEG A; 4 out of 6 for SEG B). Thus, the above-described method to verify the representativeness of the SEG and the statistical test for comparison with the OELVs cannot be applied. Based on the obtained results, it is only possible to state that occupational exposure of the monitored workers was always considerably lower than the OELV, in both SEGs: in fact, exposure values at least lower than 1/100th of the OELV are observed. In this regard, it should be noted that, according to the EN 482 standard, an analytical method should be able to measure 1/10 of the reference value with a measurement uncertainty ≤ 50%. Furthermore, according to the EN 689 standard, for an occupational exposure assessment, it is necessary to be able to measure exposures lower than 1/10 of the limit value. The analytical method used respects these requirements and the results obtained can be considered valid (LOQ for a sample of 0.4 m^3^ = 0.039 µg/m^3^; OELV = 5 µg/m^3^; LOQ/VLEP percentage ratio = 0.78%). With regards to the periodic re-assessment, the criterion defined by Appendix I of the EN 689 standard cannot be applied. However, since the mean of the measured occupational exposure was less than one-tenth of the OELV for both the SEGs, the maximum time interval before a subsequent evaluation can be fixed at 36 months.

Beyond compliance with the OELV for Cr (VI), which in this case study does not present any critical issues, it has been demonstrated that the NIOSH 7600 method systematically provides widely overestimated values of Cr(VI) airborne concentrations and occupational exposure, if compared with the duplicated samples analyzed through the ISO-modified extraction protocol. As the main goal of this work is the development of a novel extraction protocol and the evaluation of its eventual weaknesses, all extractions were intentionally carried out without the addition of the Fe(II)/Fe(III) redox buffer, to assess if its utilization is really necessary or not. In this respect, we can see that, from the data reported in Table 5, 4 out of 12 samples (A3, A4, B4, and B5) show a significant reduction of the ^50^Cr(VI) spike, together with quite low ORP values. This evidence clearly indicates that the utilization of the Fe(II)/Fe(III) buffer should be considered mandatory for the developed extraction protocol.

## 4. Conclusions

The proposed protocol for total Cr(VI) airborne determination has been proven to be very robust in the challenging case study of leather industries, where Cr(VI) has to be determined in the presence of huge concentrations of Cr(III). In particular, the development of a novel separation method capable of detecting Cr(VI) with high sensitivity and selectivity, while also tolerating high Cr(III) concentrations, highlights the merits of LC-ICP-MS as a potential future methodology to analyze Cr(VI) also in other matrixes. 

The utilization of the isotopic spiking technique was fundamental both to study the problems of the NIOSH method and to assess the goodness of the developed extraction procedure here reported. Apart from the method development, the isotopic spiking technique is recommended for the analysis of all samples as all determination becomes automatically validated and self-consistent.

The measurement of the ORP values was revealed to be a very good indicator of the Cr(III)/Cr(VI) stability during extraction and will be used by our research group to extend the applicability of the developed protocol to other industrial fields. In this context, the addition of the Fe(II)/Fe(III) redox buffer is very efficient in preventing Cr(VI) reduction induced by coextracted substances and it is supposed to work well even in the presence of oxidizing coextracted substances. 

## Data Availability

Not applicable.

## Supplementary Materials

The following supporting information can be downloaded at: https://www.mdpi.com/article/10.3390/ijerph191912111/s1, Section S1: Natural Cr(VI) and isotopic ^50^Cr(VI)-^53^Cr(III) spikes quantifications; Section S2: Environmental Monitoring; Section S3: EN 689 strategy for testing compliance with OELV; Section S4: Gravimetric results of the inhalable particle fraction. Reference [46] is cited in the supplementary materials.
