# Supplementary material for: On the Determination of Cr(VI) in Cr(III)-Rich Particulates: From the Failure of Official Methods to the Development of an Alternative Protocol
Source: Int J Environ Res Public Health. 2022 Sep 24;19(19):12111. doi: 10.3390/ijerph191912111 (PMC9564694; doi:10.3390/ijerph191912111)
Supplement: Supplementary file 1 [file ijerph-19-12111-s001.zip › ijerph-1928120-supplementary.pdf]

Supplementary Materials

Article

On the determination of Cr(VI) in Cr(III)-rich particulates: from the failure of official methods to the development of an alternative protocol

Andrea Spinazzè [1,*], Davide Spanu [1], Pietro Della Bella [1], Cristina Corti [2], Francesca Borghi [1], Giacomo Fanti [1], Andrea Cattaneo [1], William Robert Wise [3], Stefan John Davis [3], Domenico Maria Cavallo [1] and Sandro Recchia [1,*]

[1] Dipartimento di Scienza e Alta Tecnologia, Università degli Studi dell'Insubria, Via Valleggio, 11, 22100 Como, Italy
[2] Dipartimento di Scienze Teoriche ed Applicate, Università degli Studi dell'Insubria, Via Dunant, 3, 21100 Varese, Italy
[3] Institute for Creative Leather Technologies, University of Northampton, University Drive, Northampton NN1 5PH, UK
* Correspondence: andrea.spinazze@uninsubria.it (A.S.); sandro.recchia@uninsubria.it (S.R.); Tel.: +39-0312-386-629 (A.S.); +39-0312-386-450 (S.R.)

S.1. Natural Cr(VI) and isotopic ^{50}Cr(VI) - ^{53}Cr(III) spikes quantifications

To determine the concentration of natural Cr(VI) and of the ^{50}Cr(VI) and ^{53}Cr(III) standard added spikes the steps here described were followed:

i. three 0-500 µg/kg calibration curves on m/z channels 50, 52 and 53 are built using natural Cr(VI) standard solutions, together with a 100 µg/kg of V (monitored on the m/z 51 channel) used as internal standard: the ratios $[signal\ nat.\ Cr/signal\ V]_{50,52,53}$ are used as ordinate values to build the calibration curves and to determine the slopes. This procedure is performed both with and without the chromatographic system in order to distinguish the signals of Cr(VI) and total Cr, respectively.
ii. The same procedure as in i) is applied using the ^{50}Cr(VI)-enriched standard solution, while for the ^{53}Cr(III)-enriched standard solution only the total chromium determination is obviously feasible.
iii. The slopes of Cr(VI) eventually deriving by the oxidation of the ^{53}Cr(III)-enriched standard solution are estimated on the basis of the ratios of the total Cr slopes determined for each m/z channel between natural Cr and ^{53}Cr(III)-enriched calibration curves: this estimation is surely valid because the ICP-MS is operated in the same conditions for both Cr(VI) and total Cr determinations.
iv. The Cr(VI) peak area signals detected on the three different 50, 52, 53 m/z channels (along with the m/z= 51 channel of V used as internal standard) can be expressed according to the three following equations:

$$\left(\frac{area_{50}}{area_{51}}\right) = \left(\left(slope_{nat\,Cr(VI)}\right)_{50} \times \left[{}^{nat}Cr(VI)\right]\right) + \left(\left(slope_{\,50Cr(VI)}\right)_{50} \times \left[{}^{50}Cr(VI)\right]\right) + \left(\left(slope_{53Cr(VI)}\right)_{50} \times \left[{}^{53}Cr(VI)\right]\right)$$

$$\left(\frac{area_{52}}{area_{51}}\right) = \left(\left(slope_{nat\,Cr(VI)}\right)_{52} \times \left[{}^{nat}Cr(VI)\right]\right) + \left(\left(slope_{\,50Cr(VI)}\right)_{52} \times \left[{}^{50}Cr(VI)\right]\right) + \left(\left(slope_{53Cr(VI)}\right)_{52} \times \left[{}^{53}Cr(VI)\right]\right)$$

$$\left(\frac{area_{53}}{area_{51}}\right) = \left(\left(slope_{nat\,Cr(VI)}\right)_{53} \times \left[{}^{nat}Cr(VI)\right]\right) + \left(\left(slope_{\,50Cr(VI)}\right)_{53} \times \left[{}^{50}Cr(VI)\right]\right) + \left(\left(slope_{53Cr(VI)}\right)_{53} \times \left[{}^{53}Cr(VI)\right]\right)$$

Where:

$\left(slope_{nat\,Cr(VI)}\right)_{xx}$ = slope of the calibration obtained with natural Cr(VI) stds on the *xx* m/z channel

$\left(slope_{50Cr(VI)}\right)_{xx}$ = slope of the calibration obtained with ^{50}Cr(VI)-enriched stds on the *xx* m/z channel

$\left(slope_{53Cr(VI)}\right)_{xx}$ = slope of the calibration estimated for ^{53}Cr(III)-enriched stds on the *xx* m/z channel

$\left[{}^{nat}Cr(VI)\right]$ = concentration of Cr(VI) determined in the analysed sample

$\left[{}^{50}Cr(VI)\right]$ = concentration of ^{50}Cr(VI)-enriched std spike recovered in the analysed sample

$\left[{}^{53}Cr(VI)\right]$ = concentration of Cr(VI) eventually formed from the ^{53}Cr(III)-enriched std spike in the analysed sample

v. The three Cr(VI) concentrations above reported are obtained implementing the three formulas reported in iv) in an excel data sheet and performing a best fit with the "solver" function (GRG non-linear): the target parameter to be optimised (to its minimum possible value) is defined by the sum of squares of the residues obtained as the differences between experimental and calculated areas.

S.2. Environmental Monitoring

The design of the monitoring campaign was defined based on the most recent technical legislation regarding the assessment of exposure to chemical agents by inhalation [1]: in particular, the general principles set out in the standard have been borrowed according to the specific objective of the research project. According to EN 689:2019 [1] a basic exposure characterization is needed to decide if personal exposure measurements are needed. For the purpose of the study, this preliminary exposure characterization of workers to various risk agents was not performed, anyway. For the aim of this study, only the exposure to Cr(VI) in a specific similar exposure group (SEG) was considered. This SEG was made up of the workers in the shaving of tanned leathers and it was specifically defined a-priori, since, as told, for this task significant levels of particulate (and therefore potential exposure to Cr) were expected. Personal exposure measurements were performed to assess the compliance

of exposure values with the adopted OELV. More broadly, the purpose of the defined procedure was to obtain valid and representative measurements of workers' exposure for comparison with the OELV. The measurement procedures followed requirements of EN 482:2021 standard [2]. Further requirements (for example regarding sensitivity, limits of quantification, specificity, sampler capacity, transport, stability, etc.) have also been verified. To measure the workers' exposure, personal sampling devices were used, placed within the breathing area of the worker (within 30 cm of the respiratory tract). The sampling duration was at least 4 hours, in compliance with EN 689 (i.e., since workers exposure may occur during the entire work shift and the assessors considered that workplace factors, including unit operations, are constant during the entire work shift, the duration of the total sampling could be less than the work period, but never less than 2 hours). Exposure measurements were carried out at two selected companies over the course of 2 working days (14-15 July 2021), during which measurements were carried out organized in double shifts for monitoring and documenting the exposure during various operations. The air temperature and relative humidity conditions of indoor work environments were also measured.

S.3. EN 689 strategy for testing compliance with OELV

At the end of the monitoring campaign and the subsequent chemical analyses, it was verified that the definition of the a-priori defined "similar exposure groups" (SEGs) complied with adequate criteria of quality, representativeness and number, for the validation of the results (EN 689 - Appendix E). When needed, and when possible, the procedure for the management of exposure data lower than the LOQ was applied (EN 689 - Appendix H), for the subsequent application of the statistical test for comparison with the OELV (EN 689 - Appendix F). The monitoring periodicity (i.e., the maximum interval between two successive assessments) was also defined on the basis of the results of the monitoring itself, according to what is defined by the same technical standard (EN 689 - Appendix I). More specifically, For the present case study, the following scheme has been defined, derived from what is defined in the UNI EN 689 standard:

i. In the event that a minority of results returned a value lower than the analytical quantification limits (LOQ), these values were treated to produce a reliable result, according to the UI EN 689 - appendix H. For the discussion in question, the "calculation method" was applied.
ii. Once the validity of each measurement has been verified (EN 689 - appendix E), it has been verified that the definition of the SEG has met adequate quality criteria, representativeness and numerousness. In particular, it was verified that the exposure data measured for the SEG had a log-normal distribution using the Kolmogorov-Smirnov test.

iii. As a last step, the statistical test was applied for the comparison with the occupational exposure limit values. The compliance can be statistically evaluated by comparing the OELV with the upper confidence limit (UCL) of 70% with the 95th percentile of the distribution of at least six measurements in Similar Exposure Group (SEG). If the UCL is lower than the OELV, it is concluded that the probability of exceeding the OELV is acceptably low: the decision is compliance. If UCL is greater than the VLPE, it is concluded that there is an unacceptable probability of risk: the decision is non-compliance. If the distribution of the results has been assumed to be log-normal, as in this case, the geometric mean (GM) and the geometric standard deviation (GSD) of the result series are calculated. For each SEG, the variable "Ur" can be calculated from the exposure data-set, according to the following formula:

$$Ur = [\ln (VLEP) - \ln (GM)] / \ln (GSD)$$

Ur is then compared to the tabulated value "Ut" as a function of the number of results. If Ur is greater than or equal to Ut, the conclusion is VLEP compliance. If Ur is less than Ut, the conclusion is non-compliance with the VLEP. In cases where the statistical test was not applicable (e.g., if the valid samples were less than 6), the preliminary test was used, as described as follows. The preliminary test requires three to five exposure measurements on workers belonging to a SEG; compliance is reached if the GM of exposure data are below:

- 0.1 × OELV for a set of three exposure measurements or,
- 0.15 × OELV for a set of four exposure measurements or,
- 0.2 × OELV for a set of five exposure measurements

iv. In case of compliance, for the purpose of defining the maximum interval for periodic measurements, the method provided by EN 689 standard (appendix I) was applied. More in detail, the calculation of the "J factor", on the basis of the geometric mean, geometric standard deviation and VL. Other than this method, more in general, if compliance is concluded, the measurements should be repeated following these criteria:

- GM < 0.1 OELV 36 months,
- 0.1 OELV < GM < 0.25 OELV 24 months,
- 0.25 OELV < GM < 0.5 OELV 18 months,
- 0.5 OELV < GM 12 months.

S.4. Gravimetric results of the inhalable particle fraction

Results of gravimetric analysis are reported below (table A-B). In all cases the determined values are far below (i.e., two order of magnitude lower) the exposure threshold values (10 mg/m^3) both for fixed-site sampling (range: 81,3 - 290 μg/m^3) and personal sampling (range: <10 - 279 μg/m^3).

Table A. Inhalable particles airborne concentrations detected with gravimetric analysis fixed-site sampling in four different shaving areas.

**Sample ID**	**Inhalable particle concentration (mg/m^3)**	
Tanned goat leather shaving	0,096	0,144
Tanned swine leather shaving	0,081	0,200
Tanned bovine leather shaving	0,200	0,290
Tanned Cr-free leather shaving	0,116	0,122
Following extraction protocol	*NIOSH*	*ISO-modified extraction protocol*

Table B. Inhalable particles occupational exposure detected with gravimetric analysis after personal sampling in two SEGs of tanned leather shavers.

**SEG**	**Sample ID**	**Inhalable particle concentration (mg/m^3)**	
A	A 1	0,297	0,161
	A 2	<0,01	<0,01
	A 3	0,062	0,040
	A 4	0,144	<0,01
	A 5	0,206	0,098
	A 6	0,134	0,160
B	B 1	<0,01	<0,01
	B 2	0,089	0,057
	B 3	0,279	0,236
	B 4	0,212	0,206
	B 5	0,087	0,080
	B 6	0,128	0,047
Following extraction protocol		*NIOSH*	*ISO-modified extraction protocol*

References

[1] UNE EN 689:2019+AC:2019 Workplace exposure - Measurement of exposure by inhalation to chemical agents - Strategy for testing compliance with occupational exposure limit values - European Standards, 2019.

[2] EN 482:2021 Workplace exposure - Procedures for the determination of the concentration of chemical agents - Basic performance requirements, (2021).
